# School‐based law enforcement strategies to reduce crime, increase perceptions of safety, and improve learning outcomes in primary and secondary schools: A systematic review

**DOI:** 10.1002/cl2.1360

**Published:** 2023-11-08

**Authors:** Benjamin W. Fisher, Anthony Petrosino, Hannah Persson, Sarah Guckenburg, Trevor Fronius, Ivan Benitez, Kevin Earl

**Affiliations:** ^1^ Department of Civil Society and Community Studies, School of Human Ecology University of Wisconsin‐Madison Madison WI USA; ^2^ WestEd Washington DC USA; ^3^ WestEd San Francisco CA USA; ^4^ Office for Safe and Healthy Neighborhoods Louisvile KY USA

## Abstract

**Background:**

School‐based law enforcement (SBLE) has become a common intervention. Although SBLE is meant to make schools safer, critics suggest it may not accomplish this purpose, and may have unintended negative consequences such as increasing students’ exclusionary discipline or contact with the criminal justice system. There may also be secondary effects related to perceptions of the school or student learning.

**Objectives:**

The purpose of this review is to synthesize the literature evaluating the use of SBLE, including outcomes related to (a) crime and behavior problems; (b) perceptions of safety; and (c) learning.

**Methods:**

We conducted a systematic literature search to identify studies that examined outcomes associated with SBLE use. Eligible studies used experimental or quasi‐experimental designs; included samples of students, teachers/staff, schools, or school districts; reported on a policing strategy focused on crime prevention or school safety that did not involve officers teaching a curriculum; included a measure that reflects crime and behavior problems, perceptions of safety, or learning; and were in a primary or secondary school. Following a multi‐stage screening process to identify studies eligible for inclusion, we estimated a series of meta‐analytic models with robust variance estimation to calculate weighted mean effect sizes for each of three main categories of outcomes and commonly occurring subsets of these categories. We examined heterogeneity in these estimates across features of the primary studies’ design.

**Results:**

The search and screening process yielded 1002 effect sizes from 32 reports. There were no true experiments, and the quasi‐experiments ranged from strictly correlational to permitting stronger causal inferences. SBLE use was associated with greater crime and behavior problems in studies that used schools as the unit of analysis. Within this category, SBLE use was associated with increased exclusionary discipline among studies that used both schools (*g* = 0.15, 95% confidence interval [CI] [0.02, 0.27]) and students (*g* = 0.003, 95% CI [0.002, 0.003]) as the unit of analysis. SBLE use was not associated with any measures of crime or violence in schools. SBLE use was associated with greater feelings of safety among studies that used schools as the unit of analysis (*g* = 0.18, 95% CI [0.13, 0.24]), although this estimate was based on only seven effect sizes from two correlational studies. All the other models, including those examining learning outcomes, yielded null results. None of the moderators tested showed meaningful relationships, indicating the findings were consistent across a variety of study design features.

**Authors’ Conclusions:**

This study's findings provide no evidence that there is a safety‐promoting component of SBLE, and support the criticism that SBLE criminalizes students and schools. Although we found no evidence of differences across methodological features, risk of bias in the primary studies limits our confidence in making causal inferences. To the extent that the findings are causal, schools that invest in strategies to improve safety will likely benefit from divesting from SBLE and instead investing in evidence‐based strategies for enhancing school safety. Schools that continue to use SBLE should ensure that their model has no harmful effects and is providing safety benefits.

## 1 PLAIN LANGUAGE SUMMARY

### 1.1 Systematic review evidence supports the criticism that school‐based law enforcement criminalizes students and schools

School‐based law enforcement (SBLE) includes a variety of forms of sworn law enforcement officers stationed in schools on at least a part‐time basis. Although SBLE is intended to maintain school safety, critics claim it is ineffective in this role and has negative consequences for students. The existing literature does not find that SBLE makes schools safer. The evidence suggests that SBLE has detrimental consequences, particularly for exclusionary discipline.

### 1.2 What is this review about?

The past several decades has witnessed a steady growth of the presence of SBLE around the world. There is no standard definition for SBLE, nor is there a common training, job description, or chain of authority. In the USA, the two most common approaches are to contract officers from a local law enforcement agency to work in the school, and school district police departments, in which the local school or district employs its own sworn police force.

There is debate about whether SBLE is effective or harmful.

This study synthesizes the empirical literature that examined the relationship between SBLE presence and school‐related outcomes including (a) crime and behavior problems; (b) perceptions of school; and (c) student learning.

**What is the aim of this review?**

The objective of this Campbell systematic review is to examine the relationship between school‐based law enforcement (SBLE) presence and school‐related outcomes.

### 1.3 What studies are included?

This review includes quantitative studies that examine any outcomes related to crime and behavior, perceptions of safety, and learning outcomes. Included studies must have compared outcomes in units with SBLE to units without SBLE (including different dosages of SBLE, such as increasing SBLE presence).

### 1.4 What are the main findings of this review?

Contrary to what is intended, schools with SBLE had higher rates of crime and behavior problems than schools without SBLE. This was primarily driven by higher rates of exclusionary discipline, with no detectable improvements to school crime or violence.

We also found that students in schools with SBLE tended to feel safer at school, although this finding is less trustworthy because it is based on very little data.

None of our other analyses showed that SBLE had beneficial effects.

These results suggest that SBLE is an ineffective practice for keeping schools safe, and even has detrimental consequences.

The findings are based on studies that vary in the extent to which we can infer causal relationships between SBLE and the outcomes of interest, so we urge caution in interpreting this as a cause‐and‐effect relationship. Still, the study's findings are consistent across studies with different methodological characteristics.

### 1.5 What do the findings of the review mean?

These findings suggest that SBLE is not an effective strategy for maintaining school safety and lead to harm for students in the form of exclusionary discipline. However, limitations in the strength of the evidence affect the ability to claim that these relationships are causal.

### 1.6 How up‐to‐date is this review?

The final electronic search for the literature included in this review was carried out on 17 July 2020.

## 2 BACKGROUND

### 2.1 The problem, condition, or issue

The past several decades has witnessed a steady growth of the presence of school‐based law enforcement (SBLE) around the world. SBLE includes a variety of forms of sworn law enforcement officers stationed in schools on at least a part‐time basis, and often on a full‐time basis. SBLE might include local police officers, sheriff's deputies, officers from school‐based police agencies, or a variety of other possible arrangements. There is no standard definition for SBLE, nor is there a common training, job description, or chain of authority. In the United States, the two most common approaches are to contract officers from a local law enforcement agency to work in the school, and school district police departments, in which the local school or district employs its own sworn police force. Although data on the number of schools using police are elusive, some estimates are available. The 2019 *Indicators of School Crime and Safety* (Musu et al., 2019) report showed that according to principal self‐reports, 42% of public schools had at least one SBLE officer present at least 1 day a week during the 2015‐2016 academic year.

Although similar figures are not available in other countries, the use of SBLE is nevertheless widespread internationally. For example, schools and school districts in Canada (Broll & Howells, 2019), Great Britain (Henshall, 2018), and Australia (Mazerolle, Bennett, et al., 2017) regularly use SBLE in one form or another. Until recently, support for police in schools—at least in the United States—has crossed political lines, with SBLE receiving political and financial support at the federal level from both major political parties (Blad, 2020; Pilkington, 2013). This support has begun to wane since the summer of 2020, following the murder of George Floyd by the Minneapolis Police Department, when school districts nationwide began to remove SBLE out of concerns about racially disparate policing practices and the negative consequences for Black students in particular.

From a law enforcement perspective, the primary intended purpose of SBLE is to maintain school safety (Community Oriented Policing Services,  n.d.). That is, SBLE should be able to prevent, mitigate, and respond to incidents of school‐based crime and violence. As law enforcement officers, they are trained to recognize and respond to threats and to conduct investigations; stationing officers with this training in school is intended to make schools safer places. This is a particular concern among the public in the United States, where high‐profile incidents of gun violence in schools have demanded responses from policymakers and school leaders alike. SBLE has often been a politically expedient response to these high‐profile incidents (Madfis, 2016; Viano et al., 2021), even while silencing or ignoring perspectives that suggest SBLE might have harmful consequences (Koon, 2020; Nolan, 2011; Turner & Beneke, 2020).

In spite of the steady growth of SBLE, criticisms of SBLE have grown louder and more pointed in recent years (Counseling Not Criminalization in Schools Act, S.2125, 2021–2022). Where it once was taken for granted that SBLE would be present in schools, many large school districts have begun the process of removing SBLE. Notable examples of this are in Toronto, Canada, and several major US cities including Minneapolis, Denver, Seattle, Oakland, and others. Calls to remove SBLE have largely focused on two criticisms: that SBLE (a) does not make schools safer, and (b) has disproportionately negative impacts on students who may already experience marginalization in school settings, including non‐White students, students with disabilities, queer students, and others. SBLE have been implicated in the so‐called “school‐to‐prison pipeline,” a process by which students of color are funneled from schools into carceral settings. Although the metaphor of a “pipeline” has its shortcomings (Crawley & Hirschfield, 2018; McGrew, 2016; Simmons, 2017), the concerns about SBLE nevertheless reflect the increasing interconnectedness of education and the criminal legal system.

## 3 THE INTERVENTION

As noted above, SBLE can take a variety of forms. Currently, the bulk of the systematically collected information about SBLE is from the United States. One nationally representative survey of school administrators—the School Survey on Crime and Safety (SSOCS)—is a biennial survey that, among other things, collects information about the presence of SBLE, their roles, and some of their characteristics. A report on the most recent survey from the 2017–2018 school year (Padgett et al., 2020) shows that among schools with SBLE presence, the SBLE were commonly involved in law enforcement‐related activities such as security enforcement and patrol (90%), motor vehicle traffic control (72%), and training school personnel in crime prevention (62%). Other common non‐law enforcement roles included recording or reporting discipline problems to school authorities (75%), mentoring students (69%) and offering law‐related teaching or training for students (36%). Additionally, this report also noted that among schools where SBLE was present, a majority of the officers carried physical restraints (95%), firearms (94%), and aerosol sprays (76%).

An additional US‐based survey of 399 SBLE officers was conducted in 2018 and provides detail about what they do and how they are trained (Education Week, 2018). For example, 41% of the officers reported that enforcing laws is their primary role, with another 17% saying that ensuring safety and security was theirs. Nearly all officers reported receiving training in responding to active shooters and conflict de‐escalation, and about three‐quarters were trained in working with youth and mental health. Only about half were trained in mentoring and working with special education students, and even fewer were trained in child trauma or the teen brain.

There is also variability in the work of SBLE across different settings. For instance, one study found that among US SBLE officers, those stationed in schools with higher levels of disadvantage were more frequently engaged in tasks related to law enforcement, whereas those stationed in less disadvantaged schools were more frequently engaged in tasks related to mentoring and education (Lynch et al., 2016). Officers’ perceptions of threats of violence also differs by setting; the Education Week (2018) survey mentioned above found that officers were more than twice as likely to believe student violence was their biggest work‐related challenge if they worked in a school with more than 50% non‐White students compared to their peers stationed in majority white schools. Related research has found that SBLE officers in a mostly white and wealthy district were primarily concerned with threats from outside the school coming in to potentially do harm, whereas officers in a racially diverse, low‐income district were more likely to see the students themselves as threats (Fisher et al., 2020).

Unfortunately, beyond the surveys mentioned here, there is little systematically collected recent information about SBLE at a national or even international scale. Still, the information that is available indicates that the roles of SBLE are varied and appear to be responsive to the school and district in which a given officer is stationed. As such, the intervention under study in this review is one with a high degree of expected heterogeneity in both its implementation and effects.

### 3.1 How the intervention might work

**Figure 1:**
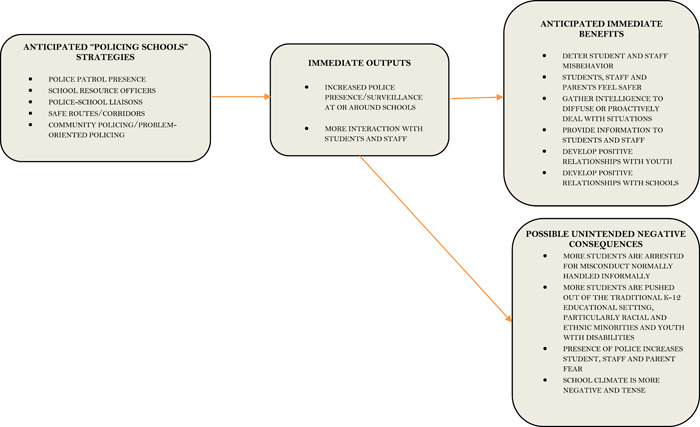
General theory of change for school‐based law enforcement programs.

A general theory of change for SBLE strategies is provided in Figure 1. As Figure 1 denotes, there are a number of interventions that fall under these strategies, including various police patrol strategies, school resource officer allocation, police‐school liaisons, safe routes/corridor programs, and community and problem‐oriented policing strategies specifically targeting the schools.

One expectation for how SBLE might work is grounded in the logic of crime deterrence (Becker, 1968; Cohen & Felson, 1979), in which crime is less likely to occur in situations where a potential offender perceives a high risk that they will be caught. In this vein, the underlying theory of SBLE programs is that they will increase police surveillance activities and interaction with students and staff at the schools. In turn, this is expected to reduce criminal behavior and non‐criminal student misconduct, increase perceptions of safety by students, staff, and parents (Theriot & Orme, 2016), and lead to more positive relationships between police with young persons and with the schools (Kupchik et al., 2020). This may also have secondary consequences that result in increased attendance, test scores and grades, and improved perceptions of the school and its climate.

However, there is possibility of unintended negative consequences that police presence leads to overuse of legal responses to behavior normally resolved by school administration (Hirschfield, 2008; Kupchik & Monahan, 2006; Simon, 2007), leading to more punishment of students (Kupchik, 2010; Nolan, 2011; Theriot, 2009), particularly among already marginalized students including racial and ethnic minorities and youth with disabilities (Homer & Fisher, 2020; Morris, 2016; Weisburst, 2019). SBLE programs could also lead to increased fear of crime if the presence of the officer sensitizes students to the possibility that violence may occur (Curran et al., 2021). Additionally, due to increased police scrutiny, it could lead to objective measures of misbehavior increasing simply because the police are on site and able to detect more student offending. These increases in surveillance, fear, punishment, and behavior problems could have a radiating effect on the school environment in which they foster negative perceptions of the school and harm learning outcomes such as grades, test scores, and attendance.

### 3.2 Why it is important to do this review

The use of SBLE is met with contrasting expectations in terms of theories of change, empirical findings, and political arguments alike. Proponents of SBLE point to the potential crime deterrent effect of SBLE and suggest that stationing officers in schools can help repair the strained relationship between law enforcement and the public. Opponents, on the other hand, contend that SBLE has a criminalizing effect on students and schools, leading to students’ increased contact with the criminal legal system. Both of these perspectives can cite theories to justify their stances and can cite empirical research that will back their claims. To help inform this debate that is occurring both in academia and the public sphere (Counseling Not Criminalization in Schools Act, S.2125, 2021–2022), this review will summarize the current body of research on SBLE, providing a quantitative synthesis of this literature. For policymakers and practitioners, this summary will provide guidance for real‐world decisions about whether and how to use SBLE. For researchers, it will corral a topic of study that spans multiple academic disciplines including Education, Criminal Justice, Sociology, and others.

## 4 OBJECTIVES

The objectives of this review were to systematically review the research to answer three research questions relevant to potential positive effects of police‐school programs, and two questions relevant to potential unintended negative consequences.

### 4.1 Potential positive effects

(1) Examine the effects of policing strategies in primary and secondary schools (K‐12 in USA) on measures of crime and non‐criminal student misbehavior (e.g., certain levels of bullying, harassment, etc.) in and around schools
(2) Examine the effects of policing strategies in primary and secondary schools on measures of staff or student perceptions of safety, climate, and culture
(3) Examine the effects of policing strategies in primary and secondary schools on learning outcomes (e.g., test scores, grades, attendance, etc.)

### 4.2 Potential negative consequences

(1) Examine the effects of policing strategies in primary and secondary schools on arrests, formal sanctions, and exclusionary discipline of students (e.g., suspensions, expulsions)
(2) Examine the extent to which these effects were disproportionate by student race/ethnicity or disability?^1^

## 5 METHODS

### 5.1 Criteria for considering studies for this review

This study has a registered protocol (Petrosino et al., 2014), although some changes were made as described below.

#### 5.1.1 Types of studies

To be included in estimates of effect, the evaluation had to include a randomized control trial or quasi‐experimental design. There are many types of quasi‐experiments (see, e.g., Shadish et al., 2002), but we included both (a) single group studies that included at least one pre‐and post‐intervention measurement, and (b) studies with a comparison group with at least one time measurement. We also included extensions of these studies that used additional measurements and/or comparison groups. Studies using interrupted time series designs were also eligible, but we identified no studies that used this design without also having a comparison group. We did not include studies that compare results from a single school to a larger pool of schools in any city or state.

#### 5.1.2 Types of participants

Studies that included samples of students, teachers/staff, individual schools, or school districts as the unit of analysis were eligible for inclusion. Given that we desired to construct as broad a search as possible to identify evaluative studies, we imposed no exclusion or inclusion criteria on the basis of the type of sample in the study. Programs may have targeted schools, districts, students, or professional staff and samples may be comprised of individuals or larger aggregate units.

#### 5.1.3 Types of interventions

To be eligible for inclusion, the document must have reported on a policing strategy focused on crime prevention or school safety goals that does not involve officers teaching a curriculum. The program had to focus on a goal such as reduced crime or increased school safety. This included strategies such as increasing the number of police patrols on school grounds. Police must either have led or have been a strong partner in the strategy. Broader programs that involved multiple agencies with minimal police involvement (e.g., police just attend a meeting of concerned agencies about a school problem) were not included. We did not include evaluations of programs in which police were among many other components (e.g., police collaborations with health and other social service agencies). In such studies, one cannot isolate the effects of the police action. The review did not include studies of the impact of academy or educational training on police performance.

There have been meta‐analyses of police‐taught curriculum such as D.A.R.E. (e.g., see Ennett, et al. 1994). The role of the officer (as a teacher) is very different in such strategies in that the focus is to deliver universal prevention strategies to students, not to gain immediate crime prevention and safety benefits at the schools. Thus, evaluations of police‐led curriculum or education programs such as D.A.R.E., G.R.E.A.T., or sexual abuse prevention curricula were not included. However, programs that involve the combination of a curriculum component with a non‐curricular policing strategy were included.

One intervention type that unexpectedly emerged during the search process was the receipt of grant funding for SBLE rather than a direct measure of SBLE presence. Studies examining the effectiveness of this funding were deemed eligible for inclusion (Owens, 2017; Weisburst, 2019).

Among studies that used comparison groups, several types of comparison groups were eligible for inclusion in this study. These included (a) groups without SBLE; (b) groups that did not add SBLE (compared to groups that did add them); (c) groups with a lower dosage of SBLE (compared to a higher dosage); and (d) groups that discontinued SBLE (compared to groups that added or retained SBLE).

#### 5.1.4 Types of outcome measures

The evaluation must have included at least one outcome measure that reflects crime, misbehavior, perceptions of safety, or student learning. This included data such as official police reports, arrests, calls for service, school disciplinary records, student self‐reports of victimization or delinquency, truancy, staff perceptions of school safety, drop out, test scores, attendance, and grades. The primary outcomes for this study are broad measures of (a) behavior, (b) perceptions of school, and (c) student learning. Secondary outcomes that emerged while conducting the study were subsets of these three primary outcomes and included: (a) school discipline; (b) violence; (c) substance‐related outcomes; (d) weapon‐related outcomes; (e) perceived safety; (f) testing and proficiency; (g) attendance; and (h) graduation.

#### 5.1.5 Duration of follow‐up

Studies using any duration of follow‐up were eligible for inclusion, including cross‐sectional studies with no follow‐up period.

#### 5.1.6 Types of settings

The policing program specifically focused on crime prevention and safety of a primary or secondary school (Kindergarten to 12th grade in US school systems). The strategy had to specifically target schools; projects that included schools in a wider community or neighborhood intervention were not eligible for inclusion in the review. Studies that tested programs implemented within primary or secondary school grounds or were focused specifically on its students (e.g., providing safe passage to school, adding school resource officers, etc.) were included. We did not include studies of policing programs implemented within preschool or higher education settings. The implications of policing preschool facilities and college/university settings are different than for policing primary/secondary schools. For example, most colleges and universities, at least in the United States, have their own police forces. In addition, students at the higher education level are adults and the campus setting involves a less controlled environment than typical K‐12 settings. In our earlier review searches, we did not come across a single study examining a police program at a preschool; this makes sense given the young age and lack of criminal intent of the population. However, elementary schools, although they also have a very young population, often are the site for school resource officer programs (e.g., Viano et al., 2021).

### 5.2 Search methods for identification of studies

This review benefited from an earlier systematic search process conducted by the WestEd Justice & Prevention Research Center for George Mason University's Center for Evidence‐based Crime Policy. The CEBCP received funding from the UK National Police Improvement Agency for a series of systematic reviews, and our proposed review of “policing schools” was selected. For that project, we searched all available years of 64 databases through December of 2009 (see Supporting Information: Appendix A), contacted 25 researchers and experts in school safety and/or school policing, and reviewed references in over 100 prior reviews and meta‐analyses (Petrosino et al., 2012).

The Campbell Collaboration review was initiated, following title registration and then protocol approval in 2014. For our searches, we examined 18 databases. Given our exhaustive searches through 2009 for the earlier review, we only examined abstracts available during 2005–2015 to provide some overlap with the earlier search process but not to be completely duplicative with those efforts. Unfortunately, many of the databases that we had access to in wave 1 were not available to us during wave 2 so there is not perfect symmetry between databases.

Because an inordinate amount of time had elapsed before we could finish the C2 editorial process, we decided to update the searches to cover more recent years to see if we found newly published studies. Those searches covered 35 databases across the years 2015–2020 (see Supporting Information: Appendix A). Again, there is not 100% symmetry as the databases we had access to during this last stage changed.

#### 5.2.1 Electronic searches

Researchers used available online resources and databases at WestEd, the University of Pennsylvania, George Mason University, Boston University, Bridgewater State University, and the University of Louisville. The databases searched can be somewhat idiosyncratic. Our approach was to conduct pilot searches of terms, working iteratively until the yield of citations and abstracts was as relevant as possible. In other words, we wanted to maximize sensitivity (getting as many citations and abstracts as possible) and specificity (making sure that as many as possible are relevant to the project).

Our search strategy combined three types of keywords. The first set of keywords (and their derivatives) targeted outcome studies: for example, “random,” “experiment,” “control,” “evaluate,” “trial,” “impact,” “effect,” and “outcome.” A second set of keywords focused the search on schools and education institutions: e.g., “student,” “school,” “district,” “classroom,” “academy,” “college,” “campus,” “teacher,” “principal,” “faculty,” “bully,” “truancy,” and “superintendent.” The third set of keywords focused the search on policing, including keywords such as “police,” “patrol,” “sheriff,” “constable,” “enforcement,” “officer,” and “security.” When the database did not permit extensive lists of keywords to be combined, simpler searches involving words such as “police” and “schools” were used. For example, a search conducted in *Criminal Justice Abstracts* was:

Query: AB = (columbine or school* or student* or campus* or teacher* or truan* or vandal* or classroom*) and AB = (experiment* or evaluat* or assess* or impact* or outcome* or effect* or randomly* or randomize* or “comparison group” or “control group” or controls* or comparisons* or “control condition” or “comparison condition” or “time series”) and AB = (police* or “law enforcement” or patrol* or policing or “security guard” or “security measure*“ or “crime prevention” or sheriff* or “cop” or “cops” or constabl* or detective* or undercover or “school crime” or “school violence” or “school safety” or “school security” or “resource officer” or “problem oriented” or Mountie*)

The exact permutations of this search term were not tracked across each individual database. The final electronic search for the literature included in this review occurred on July 17, 2020.

#### 5.2.2 Searching other resources

In addition to the electronic search, we used five other search strategies to supplement this study's literature search.

First, we identified prior review pieces and reviewed their references. Besides the aforementioned Fisher and Hennessy (2016) and Shaw (2004) reviews, there are systematic and narrative reviews of related topics that may have captured studies eligible for inclusion in this project. For example, reviews of research on the effects of strategies to reduce school violence (e.g., Derzon & Wilson, 1999; Mytton et al., 2006) could include police‐involved strategies if they met other criteria for those reviews. We identified reviews, retrieved those documents, and inspected the citations in these reviews.

Second, in an effort to be comprehensive in our electronic search strategy, we used a series of Google searches. Many institutions are putting their evaluation reports on the World Wide Web and these may not appear in peer‐reviewed journals that are indexed in electronic databases. A well‐crafted Google search strategy can uncover some evaluation reports that did not get indexed into the bibliographic databases and websites mentioned above. Although we acknowledge that Google's algorithms do not allow for replicable searches over time, we deemed this an important step for our goal of comprehensiveness.

Third, the reference section of every retrieved evaluation report was also checked to determine whether any possible eligible evaluations were listed. As noted in the eligibility criteria, we did not exclusively seek English language reports. There are also a number of general articles on policing and schools (e.g., Shaw, 2004) that reference evaluative studies, and all relevant citations from these papers were checked.

Fourth, we contacted the “informal college” of researchers on this topic and professional associations of practitioners. There is an active network of researchers conducting or aware of evaluative studies on this topic. We reached out to such researchers by email to query them about studies that may be relevant to our project. We also contacted directors of professional associations of practitioners in education and juvenile justice, including the International Association of Chiefs of Police (IACP) and the National Association of School Resource Officers (NASRO). We asked our colleagues from other nations for help in identifying any non‐English studies. WestEd also has employees who are bilingual in Spanish, French, Japanese, and Chinese and can translate abstracts or full‐text documents in non‐English to determine their eligibility for this search project. No reports were uncovered during the search, however, that required translation.

### 5.3 Data collection and analysis

#### 5.3.1 Description of methods used in primary research

The research included in this study uses quasi‐experimental designs that include some sort of comparison condition. Studies that meet these design requirements may vary widely, but generally follow a few general designs. In the simplest design, cross‐sectional studies may compare outcomes across units that had or did not have SBLE. More complex designs include two‐group, two‐wave studies that examine change over time in a treatment group compared to change over time in a comparison group. Even more complex designs may incorporate multiple waves before and/or after the introduction of SBLE. Additionally, some studies try to equate the treatment and comparison conditions through strategies like matching, using covariates, propensity score methods, or other strategies. Still other studies use fixed effects to estimate within‐unit change associated with a change in exposure to SBLE.

As noted above, a wide range of outcome variables are eligible for inclusion in this study, provided that they fit into the broad domains of (a) crime and behavior; (b) perceptions of school; and (c) learning outcomes.

#### 5.3.2 Criteria for determination of independent findings

Each report deemed eligible for inclusion was examined for the extent to which it was independent of other included reports. Reports were determined to be non‐independent if they contained identical statistical models presented across more than one report. This occurred with one dissertation (Sullivan, 2013) and a peer‐reviewed article resulting from it; in this case, we retained the dissertation that contained more analyses and a more thorough description of the methods.

The widespread use of data from the School Survey on Crime and Safety (SSOCS)—a nationally representative biennial survey of US public schools—presented a challenge in this study. One approach could have been to cluster all studies that used SSOCS data together to account for their nonindependence due to using data from the same study. However, there was enough heterogeneity in both (a) the waves of SSOCS used in the analysis, and (b) study designs that we decided to treat each report as an individual and use moderation analysis to examine whether the mean weighted effect sizes were significantly different in studies that used the SSOCS compared to those that did not. As an example of the extent to which the but the studies often used different approaches including using a single wave of data (Swartz et al., 2016), creating two‐wave panel data using a subset of schools from three waves of data (Na & Gottfredson, 2013), creating two‐wave panel data using a subset of schools from four waves of data (Devlin & Fisher, 2021), and as outcomes in a study leveraging instrumental variables (Owens, 2017).

#### 5.3.3 Selection of studies

**Figure 2:**
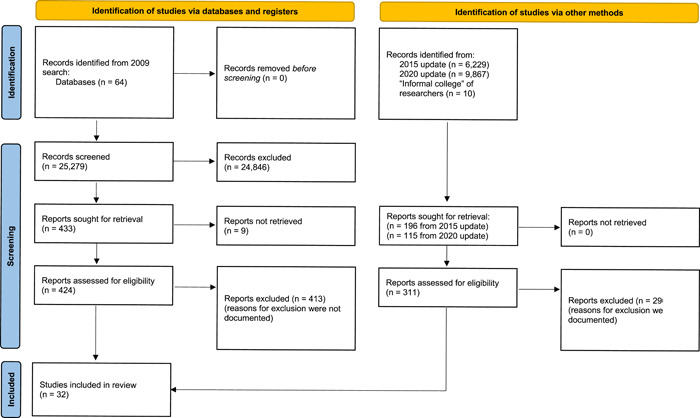
PRSIMA study search and screening flow diagram.

Studies were screened for inclusion using a multi‐stage process. The first stage was a title and abstract screening, in which members of the research team read the titles and abstracts, excluding any reports that were clearly off‐topic. Next, team members conducted a full‐text screening, verifying that each report met all the eligibility criteria for inclusion, excluding any reports that did not meet these criteria (see Figure 2). All reports reviewed in the full‐text screening were reviewed by two team members, and any disagreements were resolved by reaching a consensus through discussion. Because consensus was the goal, we did not conduct interrater reliability.

#### 5.3.4 Data extraction and management

Our data extraction followed different processes with the studies collected in the 2009 and 2015 searches compared to the data collected in the 2020 search. As part of the registered protocol, we designed a preliminary instrument to guide us in extracting information from each study (see Supporting Information: Appendix B) that was applied to all the studies deemed eligible from the 2009 and 2015 searches. Two coders^2^ reviewed each study along with the data extraction instrument and entered their findings into a spreadsheet that were compared with the other coder's findings. Any disagreements were resolved by reaching a consensus through discussion. The instrument evolved during the data extraction process (specifically regarding the information about study designs extracted from each report), and coders revisited previously coded studies to extract any new information. Although the instrument contained several open‐ended items, these were collapsed when appropriate into a smaller number of categories to permit further analysis. The instrument included items in multiple areas outlined below.

First, it included information on *researcher, study, and contextual characteristics*. Study reports can be used to provide information about the publication and characteristics about the study and its context. For example, we extracted data about the type of publication the study was reported in and the setting in which the study was conducted. If the documents provided information on the context in which the study takes place, we included it.

Second, it included information on *study methods and methodological quality*. This included features of the effect size and the study as a whole: (a) whether an effect size estimate was based on cross‐sectional or longitudinal data; (b) whether the effect size estimate controlled for a lagged measure of the outcome; (c) the number of control variables included in the estimation of the effect size; and (d) whether the study used data from the School Survey on Crime and Safety, a common source of data in this field of research.

Third, it included information on *participants in the study*. These items solicited detail about the type of participants in the studies, including information on the unit of analysis, country where the study took place, and the school level targeted. Several additional data elements pertaining to the study participants were coded after the initial search but were dropped during the 2020 update because there had been so much missing data in the studies already coded.

Fourth, it included information on *outcomes*. For each eligible study (each study has, at minimum, one outcome measure meeting the eligibility criteria described earlier), we extracted information on reported outcomes of crime and other behavior, perceptions of school, and learning outcomes. Each effect size was placed into one of these three categories, which were reviewed post hoc for common measures that could be grouped together.

Following the updated 2020 literature search, the data collection instrument was greatly reduced because very few studies from the prior searches reported relevant data. For example, school racial composition was rarely reported in the primary studies. Thus, when extracting data from studies identified in the 2020 update, we focused on coding (a) effect sizes, and (b) information about the study design. Due to limited funding, only one person coded the data extracted from the studies identified in the 2020 update.

#### 5.3.5 Assessment of risk of bias in included studies

Given that studies in this field of research do not use random assignment, selection bias is a particularly salient form of potential bias. As such, we extracted information about each effect size to gauge the extent to which the estimation of the effect size may have mitigated the potential impact of selection bias or other threats to internal validity. Although there are commonly used tools for assessing study‐level risk of bias, those instruments are not included here because the unit of analysis is the effect size, not the study. We drew multiple effect sizes from each study, and the effect sizes from a single study do not always leverage the same set of methodological characteristics. For example, if a study presented both a correlation matrix and an adjusted regression table, we included both of these effect sizes even though they do not benefit from the same methodological features of the study. So, the risk of bias for one of these effect sizes (i.e., the correlation) is greater than the other (i.e., the adjusted regression coefficient). To address this, we collected information on three different methodological characteristics of each individual effect size and used those measures in moderator analyses. First, we assessed whether the effect size was based on longitudinal or cross‐sectional data. Cross‐sectional data are more likely to have an ambiguous time order in the estimation of the effect size. Second, we assessed whether the effect size controlled for prior values of the outcome. Controlling for prior values of the outcome allows for the examination of within‐unit effects rather than comparing between treated and untreated units which is more likely subject to selection bias. Third, we assessed how many control variables were included in the calculation of each effect size. Estimates that include more control variables are likely to explain more variance in the outcome and are less likely to mistakenly attribute some of this variance to the focal predictor (in this case, SBLE presence).

#### 5.3.6 Measures of treatment effect

The summary effect size used in this study was the standardized mean difference. The standardized mean difference captures information about the difference in the mean (of the outcome variable) across two groups, which in this case included units with and without exposure to SBLE. Although Cohen's *d* is perhaps the most common measure of the standardized mean difference, all effect sizes were transformed to Hedges’ *g* before the meta‐analysis because of its favorable statistical properties for synthesis (Borenstein et al., 2009).

#### 5.3.7 Unit of analysis issues

The studies eligible for inclusion in this review were performed at multiple units of analysis (e.g., school‐level; student‐level). As such, we conducted separate analytic models to synthesize studies that used the same unit of analysis. That is, effect sizes that used one unit of analysis (e.g., schools) were not combined with effect sizes that used another unit of analysis (e.g., students).

#### 5.3.8 Dealing with missing data

Issues with missing data arose in the calculation of the effect sizes; sometimes reports did not include enough information to calculate an effect size. To address this, we used a variety of methods to obtain the necessary information. In some instances, we contacted authors to provide supplemental information. When this was an ineffective strategy, we used other means. For example, if we were missing information on the standard deviation of the dependent variable, we used the standard deviation from a similar dependent variable from a different study. Another example is that if we needed information about the number of students in a school, we referred to the US Department of Education's Common Core of Data to retrieve that information. Using these estimates and approximations allowed for a variety of effect sizes to be included in the study that might otherwise have been dropped.

#### 5.3.9 Non‐standard designs

When clustering occurred in studies that used students as the unit of analysis but studied an intervention that happened at the school level, we used all effect sizes from the studies, including those that accounted for clustering (e.g., regression coefficients from models that included school fixed effects) and those that did not (e.g., correlations). When hand calculating effect sizes from these studies, there was never enough information to account for the clustering of students in schools, so we calculated differences between either (a) students in treatment versus comparison schools, or (b) pre‐ and post‐intervention measurements within treatment schools.

#### 5.3.10 Assessment of heterogeneity

We examined heterogeneity in the meta‐analytic models using two statistics. First, we used *τ* ^*2*^, which represents the between‐study variability of the mean weighted effect sizes and is presented in the metric of the effect size itself. Second, we used the *I* ^2^ statistic, which represents the percent of heterogeneity detected that is attributable to real heterogeneity rather than sampling error.

#### 5.3.11 Assessment of reporting biases

Publication bias is a common threat to validity in meta‐analysis. Most instruments for examining publication biases in meta‐analysis are designed for syntheses that include one effect size per study. However, the current study uses multiple effect sizes from each study, making the use of these instruments questionable. Nevertheless, we used three strategies for understanding the extent to which publication bias may be present in the current study, each without accounting for the correlated effect sizes. First, we conducted a visual examination of funnel plots that plot the effect size against the standard error of the effect size. Asymmetry in the funnel plot—particularly an absence of data points in the bottom left of the graph—is an indicator that publication bias may be present. Second, we used Egger's regression test. This extends the visual examination of a funnel plot to calculate a trendline among the data points. A statistically significant coefficient indicates the presence of publication bias. Third, we used a trim‐and‐fill analysis that sequentially “trims” extreme values from the funnel plot and “fills” a data point that is equidistant from the estimated mean effect size, but in the opposite direction. This iterative process continues until plot symmetry is achieved, and the greater number of effect sizes trimmed and filled indicates a greater amount of publication bias.

#### 5.3.12 Data synthesis

The main estimation strategy used in the meta‐analysis was robust variance estimation (Hedges et al., 2010; Tipton, 2013) using the *robumeta* package in R (Fisher et al., 2017). This strategy accounts for correlated effect sizes within reports and corrects the estimates accordingly. This is important because if one report contains one effect size and another report contains 50, the latter report should not contribute 50 times as much information to the meta‐analytic model. We used correlated effects models with an assumed correlation of 0.80. We did not make any small‐sample adjustments when implementing these models.

Robust variance estimation meta‐analytic models were applied to six primary models. The six primary models included separate estimates of school‐level and student‐level effect sizes for three broad sets of outcomes: (a) crime and behavior outcomes; (b) perception outcomes; and (c) learning outcomes. Additionally, after extracting all the data from the primary studies, we grouped types of measures that were particularly common, subsetted the data to isolate these common measures, and estimated robust variance estimation models using only these data. The different subsets included: (a) discipline, (b) criminal justice system contact, (c) violence, (d) substance‐related outcomes, (e) weapon‐related outcomes, (f) perceived safety, (g) testing and proficiency, (h) attendance, and (i) graduation.

#### 5.3.13 Subgroup analysis and investigation of heterogeneity

To investigate any heterogeneity in the six primary models, we estimated a series of meta‐regression models, one for each of the six primary models. These meta‐regression models included up to four moderators in each model: (a) an indicator of whether the effect size was based on a cross‐sectional estimate; (b) an indicator of whether the effect size controlled for prior values of the outcome; (c) whether the effect size came from a published report; (d) whether the effect size was derived from the School Survey on Crime and Safety (SSOCS; a common data source among the included reports); and (e) the number of control variables used in the estimation of the effect size. When the moderators were not measured or had no variability within the effect sizes in each of the six models, they were dropped from the meta‐regression. Some of the models with a small number of effect sizes were under‐powered to detect moderation, making Type 2 errors particularly likely in these models.

#### 5.3.14 Sensitivity analysis

In meta‐analytic models that use robust variance estimation for correlated effect sizes, information is needed about *ρ*, the value of the intercorrelations among the correlated effect sizes. This information is not commonly reported in primary studies, so meta‐analysts must supply an estimate. We used a value of 0.80 but assessed the extent to which the model estimates were sensitive to this choice by examining *ρ* values ranging from 0.00 to 1.00 in increments of 0.2.

#### 5.3.15 Treatment of qualitative research

Qualitative research was not included in this review.

## 6 RESULTS

### 6.1 Description of studies

#### 6.1.1 Results of the search

The flow diagram of study screening and selection is included in Figure 2. The original search that was conducted in 2009 was done across 64 databases and yielded 25,279 records for screening. After screening titles and abstracts, 424 records remained and underwent a full‐text review. Following this review, 11 studies were eligible for inclusion. Two separate search updates occurred in the following years: one in 2015 and one in 2020. These two searches yielded 6229 and 9867 records, respectively, and were supplemented with 10 records from the “informal college” of researchers. Among these studies, 311 underwent a full‐text review that identified 21 studies eligible for inclusion. This yielded a final sample of 1002 effect sizes from 32 studies.

#### 6.1.2 Included studies

Table 1 displays key characteristics of the included studies. Of the 1002 effect sizes in the meta‐analysis, 42.6% were derived from published studies. Among all the effect sizes, 520 examined crime and behavior outcomes using schools as the unit of analysis; 349 examined crime and behavior outcomes using students as the unit of analysis; 13 examined perception outcomes using schools as the unit of analysis; 20 examined perception outcomes using students as the unit of analysis; 41 examined learning outcomes using schools as the unit of analysis; and 56 examined learning outcomes using students as the unit of analysis. Additionally, 406 effect sizes were derived from cross‐sectional estimates (note that several studies contributed effect sizes that were calculated from longitudinal and cross‐sectional data), 216 controlled for lagged values of the outcome, and 88 came from the SSOCS. On average, the effect sizes were calculated with adjustments for between 4 and 5 control variables, although over half included zero controls. All effect sizes were based on data that included secondary (i.e., middle and/or high) schools at least in part. All but three of the studies were based in the United States.

**Table 1:** Characteristics of included studies.

Citation	Document type	Country of study	Unit of analysis	Sample size^a^	Number of ES contributed to Behavioral Outcomes analysis	Number of ES contributed to Learning Outcomes analysis	Number of ES contributed to Perception Outcomes analysis
Anderson (2018)	Journal Article	United States	Schools	471	26	0	0
Barnes (2008)	Dissertation	United States	Schools	240	5	0	0
Bhabra et al. (2004)	Technical Report	United Kingdom	Students	1175	5	0	10
Boman and Mowen (under review)	Unpublished Manuscript	United States	Schools	653	4	0	0
Bowles et al. (2005)	Technical Report	United Kingdom	Schools	30	4	10	0
Brady et al. (2007)	Journal Article	United States	Schools	20	8	4	0
Devlin and Fisher (2021)	Journal Article	United States	Schools	850	24	0	0
Devlin and Gottfredson (2018)	Journal Article	United States	Schools	480	12	0	0
Devlin, and Santos, and Gottfredson (2018)	Journal Article	United States	Schools	480	10	0	0
Dohy and Banks (2018)	Journal Article	United States	Schools	148	2	0	0
Fisher (2016)	Dissertation	United States	Schools	162	1	0	0
Fisher and Devlin (2020)	Journal Article	United States	Schools	850	30	0	0
Gottfredson et al. (2020)	Journal Article	United States	Schools	105	72	0	0
Homer and Fisher (2020)	Journal Article	United States	Schools	92,620	18	0	0
Katz et al. (2002)	Technical Report	United States	Students	5816	56	4	10
Maguire et al. (2003)	Technical Report	United States	Schools	2	28	3	0
McKay et al. (2006)	Technical Report	Canada	Schools	4	15	1	5
Miller (1968)	Technical Report	United States	Schools	8	55	1	0
Na and Gottfredson (2013)	Journal Article	United States	Schools	470	11	0	0
Owens (2017)	Journal Article	United States	Schools	Multiple samples used	16	0	0
Rogers (2004)	Dissertation	United States	Schools	19	0	2	0
Scanlon (2016)	Dissertation	United States	Schools	2517	24	12	0
Sorensen et al. (2021)	Journal Article	United States	Schools	450	75	8	0
Stevenson (2011)	Dissertation	United States	Schools	18	15	0	0
Stokes et al. (1996)	Technical Report	United States	Schools	4	4	0	8
Sullivan (2013)	Journal Article	United States	Schools and students	48,636	216	8	0
Swartz et al. (2016)	Journal Article	United States	Schools	1699	1	0	0
Templeman (1979)	Technical Report	United States	Schools	26	3	0	0
Theriot (2009)	Journal Article	United States	Schools	28	7	0	0
Weisburst (2019)	Journal Article	United States	Students	2,506,849	77	44	0
Zhang (2019)	Journal Article	United States	Schools	238	48	0	0

^a^ In cases in which multiple effect sizes were drawn from a single study, the sample size may differ across effect sizes.

#### 6.1.3 Excluded studies

A list of studies excluded at the full‐text review stage is included in the References. These studies were excluded for a variety of reasons, including being non‐empirical studies, using qualitative methods, not including police in schools, and other reasons. These reasons for exclusions are listed in Figure 2.

### 6.2 Risk of bias in included studies

The risk of bias in the included studies was high. There were no studies that used random assignment, so our assessment of risk of bias was based on the methodological features of the quasi‐experiments, looking in particular for features of the study design or analyses that would improve both internal and external validity. We extracted effect size estimates that were derived from a variety of designs, ranging from cross‐sectional studies without any controls to difference‐in‐difference designs with a robust set of controls to help mitigate the potential impact of selection bias. Several studies used rigorous quasi‐experimental designs to estimate the causal impact of SBLE on outcomes of interest. For example, two studies (Owens, 2017; Weisburst, 2019) used instrumental variable approaches with credibly exogenous variation in the implementation of SBLE. Another study (Gottfredson et al., 2020) used a comparative interrupted time series design with observations at 43 time points. Additionally, five studies (Anderson, 2018; Devlin & Santos, & Gottfredson, 2018; Owens, 2017; Scanlon, 2016; Sorensen et al., 2021) used a difference‐in‐differences framework to examine change over time relative to a comparison group. Another seven studies (Barnes, 2008; Devlin & Fisher, 2021; Devlin & Gottfredson, 2018; Dohy & Banks, 2018; Fisher, 2016; Fisher & Devlin, 2020; Na & Gottfredson, 2013) used either student fixed effects with student‐level data or school fixed effects with school‐level data to examine within‐unit change over time, or controlled for a lagged measure of the outcome. Additionally, four other studies (Brady et al., 2007; Homer & Fisher, 2020; Swartz et al., 2016; Zhang, 2019) applied matching or weighting techniques to reduce selection bias. The remaining studies did not include elements of either their design or analysis that permitted strong causal inferences.

There was also variability in the external validity of the included studies. Some were very strong, including three studies that used nationally representative data from the United States (Boman & Mowen, under review; Homer & Fisher, 2020; Swartz et al., 2016). Seven other studies with particularly strong external validity used statewide data from a single US state (Anderson, 2018; Barnes, 2008; Fisher, 2016; Sorensen et al., 2021; Sullivan, 2013; Weisburst, 2019; Zhang, 2019). Another six studies drew on data that were nationally representative in their original form, but used a subset of data that made the data no longer nationally representative (Devlin & Fisher, 2021; Devlin & Gottfredson, 2018; Devlin & Santos, & Gottfredson, 2018; Fisher & Devlin, 2020; Na & Gottfredson, 2013; Owens, 2017). The remaining studies had weak external validity, focusing on a set of schools that was not evidently representative of a broader population.

### 6.3 Synthesis of results

The results from the series of meta‐analytic models are presented in Table 2 As shown, for each set of outcomes, models were estimated separately for effect sizes that were based on studies that used either students or schools as the unit of analysis.

**Table 2:** Weighted mean effect size estimates.

	*g*	SE	*p*	LB	UB	*τ* ^2^	*I* ^2^	*k*	*n*
*Crime and behavior outcomes*									
Overall									
School‐level	0.09	0.04	0.023	0.01	0.16	0.08	95.88	27	520
Student‐level	−0.09	0.17	0.657	−0.86	0.69	0.27	95.57	4	349
Discipline									
School‐level	0.15	0.06	0.025	0.02	0.27	11.00	126.00		
Student‐level	0.003	0.000	0.008	0.002	0.003	0.00	0.00	2	73
Criminal justice system contact									
School‐level	0.03	0.03	0.365	−0.05	0.11	0.01	81.03	7	82
Student‐level	—	—	—	—	—	—	—	—	—
Violence									
School‐level	0.09	0.07	0.214	−0.06	0.24	0.10	92.49	18	91
Student‐level	−0.02	0.15	0.919	−1.88	1.84	44.30	97.44	2	46
Substance‐related outcomes									
School‐level	0.04	0.05	0.404	−0.08	0.17	0.42	97.72	8	45
Student‐level	−0.23	0.30	0.582	−4.06	3.59	3.55	97.83	2	56
Weapon‐related outcomes									
School‐level	−0.04	0.14	0.772	−0.36	0.28	0.44	97.24	10	35
Student‐level	0.05	0.01	0.160	−0.12	0.23	13.63	75.71	2	31
*Perception outcomes*									
Overall									
School‐level	0.06	0.08	0.624	−1.01	1.12	0.95	62.09	2	13
Student‐level	0.11	0.05	0.269	−0.51	0.72	6.19	66.24	2	20
Perceived safety									
School‐level	0.18	0.00	0.016	0.13	0.24	0.68	55.55	2	7
Student‐level	−0.03	0.04	0.585	−0.53	0.47	2.23	43.11	2	12
*Learning outcomes*									
Overall									
School‐level	−0.07	0.07	0.422	−0.32	0.18	0.03	22.94	8	41
Student‐level	0.37	0.38	0.448	−1.73	2.48	132.72	90.04	3	56
Testing and proficiency									
School‐level	−0.03	0.05	0.606	−0.48	0.42	0.01	21.24	3	18
Student‐level	—	—	—	—	—	—	—	—	—
Attendance									
School‐level	−0.22	0.32	0.539	−1.17	0.73	0.27	60.44	5	16
Student‐level	—	—	—	—	—	—	—	—	—
Graduation									
School‐level	—	—	—	—	—	—	—	—	—
Student‐level	—	—	—	—	—	—	—	—	—

Abbreviations: *g*, Hedges’ *g*; *k*, number of reports; LB, lower bound of 95% confidence interval; *n*, number of effect sizes; SE, standard error; UB, upper bound of 95% confidence interval.

#### 6.3.1 Crime and behavior

In the model synthesizing all the crime and behavior outcomes that used schools as the unit of analysis, there were 520 effect sizes that came from 27 studies. The robust variance estimation model with a *ρ* value of 0.8 indicated that the mean weighted effect size was 0.09 (*p* = 0.023, 95% confidence interval [CI] [0.01, 0.16]). This estimate was statistically significantly different from zero, indicating that schools with SBLE had more problems with crime and behavior than schools without SBLE. This effect is in the opposite direction of what would be expected if SBLE had a crime deterrent effect. There was also substantial heterogeneity between studies in this model, with a relatively large *τ* ^*2*^ value of 0.08. Moreover, the *I* ^2^ value of 95.88 indicates that this heterogeneity is nearly all true heterogeneity and not a result of sampling error.

In the model synthesizing all the crime and behavior outcomes that used students as the unit of analysis, there were 349 effect sizes that came from four studies. The robust variance estimation model with a *ρ* value of 0.8 indicated that the mean weighted effect size was −0.09 (*p* = 0.657, 95% CI [−0.86, 0.69]). This estimate was not statistically significantly different from zero, indicating that students in schools with SBLE had statistically indistinguishable problems with crime and behavior compared to students in schools without SBLE. There was also substantial heterogeneity between studies in this model, with a relatively large *τ* ^*2*^ value of 0.27. Moreover, the *I* ^*2*^ value of 95.57 indicates that this heterogeneity is nearly all true heterogeneity and not a result of sampling error.

##### Discipline

The first subgroup analysis focused only on outcomes related to school discipline. In the model synthesizing effect sizes that used schools as the unit of analysis, there were 126 effect sizes that came from 11 studies. The robust variance estimation model with a *ρ* value of 0.8 indicated that the mean weighted effect size was 0.15 (*p* = 0.025, 95% CI [0.02, 0.27]). This estimate was statistically significantly different from zero, indicating that schools with SBLE had higher rates of discipline than schools without SBLE. This effect is consistent with what would be expected by the school criminalization perspective, with more punishment occurring in schools with SBLE. This model had substantial heterogeneity between studies (*τ* ^2^ = 0.16), nearly all of which was true heterogeneity (*I* ^2^ = 91.78).

In the model synthesizing effect sizes that used students as the unit of analysis, there were 73 effect sizes that came from two studies. The robust variance estimation model with a *ρ* value of 0.8 indicated that the mean weighted effect size was 0.003 (*p* = .008, 95% CI [0.002, 0.003]). This estimate was statistically significantly different from zero, indicating that students in schools with SBLE experienced more discipline than students in schools without SBLE, although the effect was very small. This is again consistent with what would be expected by the school criminalization perspective. This model detected no heterogeneity between studies (*τ* ^2^ = 0.00; *I* ^2^ = 0.00).

##### Criminal justice system contact

The second subgroup analysis focused only on outcomes related to criminal justice system contact. In the model synthesizing effect sizes that used schools as the unit of analysis, there were 82 effect sizes that came from seven studies. The robust variance estimation model with a *ρ* value of 0.8 indicated that the mean weighted effect size was 0.03 (*p* = 0.365, 95% CI [−0.05, 0.11]). This estimate was not statistically significantly different from zero, indicating that schools with SBLE had statistically indistinguishable rates of criminal justice system contact compared to schools without SBLE. This model had a small amount of heterogeneity between studies (*τ* ^2^ = 0.01), most of which was true heterogeneity (*I* ^2^ = 81.03).

Only one report included effect sizes pertaining to criminal justice system contact with students as the unit of analysis, so no meta‐analytic models were estimated.

##### Violence

The third subgroup analysis focused only on outcomes related to violence. In the model synthesizing effect sizes that used schools as the unit of analysis, there were 91 effect sizes that came from 18 studies. The robust variance estimation model with a *ρ* value of 0.8 indicated that the mean weighted effect size was 0.09 (*p* = 0.214, 95% CI [−0.06, 0.24]). This estimate was not statistically significantly different from zero, indicating that schools with SBLE had statistically indistinguishable rates of violence compared to schools without SBLE. This model had a substantial amount of heterogeneity between studies (*τ* ^2^ = 0.10), nearly all of which was true heterogeneity (*I* ^2^ = 81.03).

In the model synthesizing effect sizes that used students as the unit of analysis, there were 46 effect sizes that came from two studies. The robust variance estimation model with a *ρ* value of 0.8 indicated that the mean weighted effect size was −0.02 (*p* = 0.919, 95% CI [−1.88, 1.84]). This estimate was not statistically significantly different from zero, indicating that students in schools with SBLE experienced statistically indistinguishable rates of violence compared to students in schools without SBLE. This model had a large amount of heterogeneity between studies (*τ* ^2^ = 44.30), nearly all of which was true heterogeneity (*I* ^2^ = 97.44).

##### Substance‐related outcomes

The fourth subgroup analysis focused only on outcomes related to substance use or possession offenses. In the model synthesizing effect sizes that used schools as the unit of analysis, there were 45 effect sizes that came from 8 studies. The robust variance estimation model with a *ρ* value of 0.8 indicated that the mean weighted effect size was 0.04 (*p* = 0.899, 95% CI [−0.08, 0.17]). This estimate was not statistically significantly different from zero, indicating that schools with SBLE had statistically indistinguishable rates of substance‐related offenses compared to schools without SBLE. This model had a substantial amount of heterogeneity between studies (*τ* ^2^ = 0.42), nearly all of which was true heterogeneity (*I* ^2^ = 97.72).

In the model synthesizing effect sizes that used students as the unit of analysis, there were 56 effect sizes that came from two studies. The robust variance estimation model with a *ρ* value of 0.8 indicated that the mean weighted effect size was −0.23 (*p* = 0.582, 95% CI [−4.06, 3.59]). This estimate was not statistically significantly different from zero, indicating that students in schools with SBLE had statistically indistinguishable rates of substance‐related offenses compared to students in schools without SBLE. This model had a large amount of heterogeneity between studies (*τ* ^2^ = 3.55), nearly all of which was true heterogeneity (*I* ^2^ = 97.83).

##### Weapon‐related outcomes

The fifth subgroup analysis focused only on outcomes related to weapon use or possession. In the model synthesizing effect sizes that used schools as the unit of analysis, there were 35 effect sizes that came from 10 studies. The robust variance estimation model with a *ρ* value of 0.8 indicated that the mean weighted effect size was −0.04 (*p* = 0.772, 95% CI [−0.36, 0.27]). This estimate was not statistically significantly different from zero, indicating that schools with SBLE had statistically indistinguishable rates of weapon‐related offenses compared to schools without SBLE. This model had a substantial amount of heterogeneity between studies (*τ* ^2^ = 0.43), nearly all of which was true heterogeneity (*I* ^2^ = 97.24).

In the model synthesizing effect sizes that used students as the unit of analysis, there were 31 effect sizes that came from two studies. The robust variance estimation model with a *ρ* value of 0.8 indicated that the mean weighted effect size was 0.05 (*p* = 0.160, 95% CI [−0.12, 0.23]). This estimate was not statistically significantly different from zero, indicating that students in schools with SBLE had statistically indistinguishable rates of weapon‐related offenses compared to students in schools without SBLE. This model had a large amount of heterogeneity between studies (*τ* ^2^ = 13.63), most of which was true heterogeneity (*I* ^2^ = 75.71).

#### 6.3.2 Perceptions of school

In the model synthesizing all the perception outcomes that used schools as the unit of analysis, there were 13 effect sizes that came from two studies. The robust variance estimation model with a *ρ* value of 0.8 indicated that the mean weighted effect size was 0.06 (*p* = 0.624, 95% CI [−1.01, 1.12]). This estimate was not statistically significantly different from zero, indicating that perceptions of schools with SBLE were statistically indistinguishable from perceptions of schools without SBLE. There was also substantial heterogeneity between studies in this model, with a relatively large *τ* ^2^ value of 0.95. Additionally, the *I* ^2^ value of 62.09 indicates that a majority of this heterogeneity is true heterogeneity, but a substantial portion is also a result of sampling error.

In the model synthesizing all the perception outcomes that used students as the unit of analysis, there were 20 effect sizes that came from two studies. The robust variance estimation model with a *ρ* value of 0.8 indicated that the mean weighted effect size was 0.11 (*p* = 0.269, 95% CI [−0.51, 0.72]). This estimate was not statistically significantly different from zero, indicating that students’ perceptions of schools with SBLE were statistically indistinguishable from students’ perceptions of schools without SBLE. There was also substantial heterogeneity between studies in this model, with a large *τ* ^2^ value of 6.19. Additionally, the *I* ^2^ value of 66.24 indicates that a majority of this heterogeneity is true heterogeneity, but a substantial portion is also a result of sampling error.

##### Perceived safety

The only subgroup analysis among the perception outcomes was perceived safety at school. In the model synthesizing all the perceived safety outcomes that used schools as the unit of analysis, there were seven effect sizes that came from two studies. The robust variance estimation model with a *ρ* value of 0.8 indicated that the mean weighted effect size was 0.18 (*p* = 0.016, 95% CI [0.13, 0.24]). This estimate was statistically significantly different from zero, indicating that perceived safety in schools with SBLE was significantly higher than in schools without SBLE. This is consistent with a framework suggesting that SBLE improves perceptions of safety at school. There was also substantial heterogeneity between studies in this model, with a relatively large *τ* ^2^ value of 0.68. Additionally, the *I* ^2^ value of 55.55 indicates that a about half of the total heterogeneity is true heterogeneity, but a substantial portion is a result of sampling error.

In the model synthesizing all the perceived safety outcomes that used students as the unit of analysis, there were 12 effect sizes that came from two studies. The robust variance estimation model with a *ρ* value of 0.8 indicated that the mean weighted effect size was −0.03 (*p* = 0.585, 95% CI [−0.53, 0.47]). This estimate was not statistically significantly different from zero, indicating that students’ perceived safety in schools with SBLE was not significantly different from students’ perceived safety in schools without SBLE. There was also a large amount of heterogeneity between studies in this model, with a *τ* ^2^ value of 2.23. Additionally, the *I* ^2^ value of 43.11 indicates that over half of the total heterogeneity was a result of sampling error, but a substantial portion is true heterogeneity.

#### 6.3.3 Learning outcomes

In the model synthesizing all the learning outcomes that used schools as the unit of analysis, there were 41 effect sizes that came from eight studies. The robust variance estimation model with a *ρ* value of 0.8 indicated that the mean weighted effect size was −0.07 (*p* = 0.422, 95% CI [−0.32, 0.18]). This estimate was not statistically significantly different from zero, indicating that learning outcomes in schools with SBLE were statistically indistinguishable from learning outcomes in schools without SBLE. There was also substantial heterogeneity between studies in this model, with a relatively large *τ* ^2^ value of 0.03. However, the *I* ^2^ value of 22.94 indicates that much of this heterogeneity is a result of sampling error and is not true heterogeneity in the effect size estimate.

In the model synthesizing all the learning outcomes that used students as the unit of analysis, there were 56 effect sizes that came from three studies. The robust variance estimation model with a *ρ* value of 0.8 indicated that the mean weighted effect size was 0.37 (*p* = 0.448, 95% CI [−1.73, 2.48]). This estimate was not statistically significantly different from zero, indicating that students in schools with SBLE had statistically indistinguishable learning outcomes from students in schools without SBLE. There was also substantial heterogeneity between studies in this model, with a very large *τ* ^2^ value of 132.72. Moreover, the *I* ^2^ value of 90.04 indicates that this heterogeneity is nearly all true heterogeneity and not a result of sampling error.

##### Testing and proficiency

The first subgroup analysis of learning outcomes addressed standardized testing and proficiency outcomes. In the model synthesizing effect sizes that used schools as the unit of analysis, there were 18 effect sizes that came from three studies. The robust variance estimation model with a *ρ* value of 0.8 indicated that the mean weighted effect size was −0.03 (*p* = 0.606, 95% CI [−0.48, 0.42]). This estimate was not statistically significantly different from zero, indicating that schools with SBLE had statistically indistinguishable standardized test scores and proficiency levels compared to schools without SBLE. This model had a small amount of heterogeneity between studies (*τ* ^2^ = 0.01), most of which was due to sampling error (*I* ^2^ = 21.24).

No reports included effect sizes pertaining to standardized testing and proficiency outcomes with students as the unit of analysis, so no meta‐analytic models were estimated.

##### Attendance

The second subgroup analysis of learning outcomes addressed attendance. In the model synthesizing effect sizes that used schools as the unit of analysis, there were 16 effect sizes that came from five studies. The robust variance estimation model with a *ρ* value of 0.8 indicated that the mean weighted effect size was −0.22 (*p* = 0.539, 95% CI [−1.17, 0.73]). This estimate was not statistically significantly different from zero, indicating that schools with SBLE had statistically indistinguishable attendance rates compared to schools without SBLE. This model had a meaningful amount of heterogeneity between studies (*τ* ^2^ = 0.27), most of which was due to true heterogeneity, although a meaningful amount was also due to sampling error (*I* ^2^ = 60.44).

Only one report included effect sizes pertaining to attendance with students as the unit of analysis, so no meta‐analytic models were estimated.

##### Graduation

The third subgroup analysis of learning outcomes addressed graduation. No reports included effect sizes pertaining to graduation with schools as the unit of analysis, and only one report included effect sizes pertaining to graduation with students as the unit of analysis, so no meta‐analytic models were estimated.

#### 6.3.4 Moderator analyses

To examine possible sources of this heterogeneity, we used meta‐regression to examine the potential role of four moderators in each of the models for the three overall outcomes (i.e., crime and behavior, perception, and learning outcomes). These four moderators included: (a) whether the effect size was based on cross‐sectional data; (b) whether the effect size controlled for prior values of the outcome; (c) whether the effect size was derived from a published (vs. unpublished) report; and (d) whether the effect size was derived from data that came from the School Survey on Crime and Safety. Moderators were dropped from a model when there was no variability in the moderator. As shown in Table 3 almost none of the moderators were statistically significant, indicating that the effect size estimates were consistent across different values of the moderators. In two models, the coefficient for the number of controls was statistically significant but had an effect so small as to be virtually meaningless—each additional control variable in the model was associated with a change in the estimated effect size of less than 0.001.

**Table 3:** Moderator analysis.

	*b*	SE	*p*	LB	UB	*τ* ^2^	*I* ^*2*^	*k*	*n*
*Crime and behavior*									
School‐level									
Intercept	0.10	0.08	0.291	−0.10	0.29	0.37	96.36	27	520
Cross‐sectional	−0.09	0.09	0.345	−0.29	0.11	0.37	96.36	27	520
Control for prior	0.03	0.10	0.786	−0.19	0.25	0.37	96.36	27	520
Published	0.14	0.08	0.108	−0.04	0.32	0.37	96.36	27	520
SSOCS	−0.03	0.07	0.704	−0.19	0.13	0.37	96.36	27	520
Number of controls	−0.01	0.00	0.093	−0.02	0.00	0.37	96.36	27	520
Student‐level									
Intercept	−0.25	0.53	0.689	−3.26	2.75	8.55	96.50	4	349
Cross‐sectional	0.10	0.60	0.881	−3.13	3.34	8.55	96.50	4	349
Published	0.27	0.53	0.676	−2.74	3.28	8.55	96.50	4	349
Number of controls	0.00	0.00	<0.001	0.00	0.00	8.55	96.50	4	349
*Perceptions*									
School‐level									
Intercept	0.00	0.00	<0.001	0.00	0.00	0.97	63.05	2	13
Cross‐sectional	0.09	0.09	0.518	−1.06	1.23	0.97	63.05	2	13
Student‐level									
Intercept	0.03	0.07	0.730	−0.92	0.98	2.64	45.98	2	20
Cross‐sectional	0.17	0.24	0.607	−2.85	3.19	2.64	45.98	2	20
*Learning outcomes*									
School‐level									
Intercept	0.13	0.30	0.437	−3.26	3.52	0.11	27.90	8	41
Cross‐sectional	−0.29	0.33	0.522	−3.66	3.07	0.11	27.90	8	41
Control for prior	−0.20	0.33	0.597	−1.61	1.21	0.11	27.90	8	41
Published	−0.76	0.17	0.068	−1.68	0.16	0.11	27.90	8	41
Number of controls	0.01	0.01	0.524	−0.10	0.13	0.11	27.90	8	41
Student‐level									
Intercept	1.02	0.83	0.434	−9.48	11.51	90.27	90.50	3	56
Cross‐sectional	−1.14	0.75	0.370	−10.65	8.37	90.27	90.50	3	56
Published	−1.07	0.83	0.418	−11.57	9.43	90.27	90.50	3	56
Number of controls	0.00	0.00	<0.001	0.00	0.00	90.27	90.50	3	56

Abbreviations: *g*, Hedges’ *g*; *k*, number of reports; LB, lower bound of 95% confidence interval; *n*, number of effect sizes; SE, standard error; UB, upper bound of 95% confidence interval.

#### 6.3.5 Publication bias

In the model for school‐level estimates of crime and behavior outcomes, there was no observable asymmetry in the funnel plot. Egger's regression test similarly was non‐significant, indicating no evidence of publication bias. The trim‐and‐fill method indicated no studies needed to be added to the plot to make it symmetric, again indicating no evidence of publication bias. In the model for student‐level estimates of crime and behavior outcomes, there was observable asymmetry in the funnel plot, but in the opposite direction of what would be expected in the presence of publication bias. Egger's regression test was significant (*p* < 0.001), but the coefficient was negative, indicating no evidence of publication bias. The trim‐and‐fill method indicated no studies needed to be added to the plot to make it symmetric, again indicating no evidence of publication bias.

In the model for school‐level estimates of perception outcomes, there was no observable asymmetry in the funnel plot. Egger's regression test similarly was non‐significant, indicating no evidence of publication bias. The trim‐and‐fill method indicated that five effect sizes needed to be added to the left side of the plot to make it symmetric, indicating that publication bias potentially inflated the estimate in this model. In the model for student‐level estimates of perception outcomes, there was no observable asymmetry in the funnel plot. Egger's regression test similarly was non‐significant, indicating no evidence of publication bias. The trim‐and‐fill method indicated seven effect sizes needed to be added to the right side of the plot to make it symmetric, the opposite side of the plot that would be expected if publication bias was present.

In the model for school‐level estimates of learning outcomes, there was no observable asymmetry in the funnel plot. Egger's regression test similarly was non‐significant, indicating no evidence of publication bias. The trim‐and‐fill method indicated that four effect sizes needed to be added to the right side of the plot to make it symmetric, the opposite side of the plot that would be expected if publication bias was present. In the model for student‐level estimates of learning outcomes, there was no observable asymmetry in the funnel plot. However, Egger's regression test was significant (*p* = 0.008), providing evidence of publication bias and suggesting that the model estimate may be inflated because of publication bias. However, the trim‐and‐fill method indicated no effect sizes needed to be added to make the funnel plot symmetric.

Overall, although these strategies for assessing publication bias are not designed for application to robust variance estimation models, they provide little evidence that publication bias was present in the six main models estimated in this study. This is perhaps not surprising given that so many effect sizes came from unpublished studies such as dissertations and technical reports.

#### 6.3.6 Sensitivity analyses

When estimating robust variance estimation models in meta‐analysis, the user must supply a value for *ρ* (the within‐study correlation among effect sizes) when it is not provided in the primary study, as was the case here. As recommended by Tanner‐Smith and Tipton (2014), we estimated models with a range of values of *ρ*, ranging from 0.0 to 1.0 in increments of 0.2. Although the results were not perfectly identical across all models, there was never an instance where the difference in estimates was greater than 0.03, which in this case is a very small difference in practical terms. Thus, the results reported here (using a *ρ* of 0.8) are consistent across a range of possible values of *ρ*.

## 7 DISCUSSION

### 7.1 Summary of main results

This study's findings indicate that SBLE programs were associated with higher levels of crime and behavior problems in studies where schools were the unit of analysis, but studies with students as the unit of analysis yielded no statistically significant differences for crime and behavior outcomes. This is particularly noteworthy given that the presumed purpose of SBLE is to deter crime and behavior problems; this study's findings provide no such evidence, and even suggest that the opposite may be true—that crime and behavior problems increase with SBLE. In examining more specific measures of crime and behavior, schools with SBLE had more discipline‐related outcomes in studies with both schools and students as the unit of analysis. There were no statistically significant differences between schools with and without SBLE for criminal justice system contact, violence, substance‐related outcomes, or weapon‐related outcomes.

Given this set of findings pertaining to outcomes related to crime and behavior, it seems that the association between SBLE and more crime and behavior problems is largely driven by outcomes related to schools’ use of exclusionary discipline such as suspensions. This is important because exclusionary discipline is not a direct measure of student behavior, but is instead a measure of schools’ responses to student behaviors. In fact, although one might assume that suspensions are reserved for only the most severe offenses, research has demonstrated that a substantial number of suspensions are administered for highly subjective offenses (e.g., disorderly conduct) or repetitive low‐level offenses (e.g., dress code violations). Moreover, research has demonstrated that schools’ use of exclusionary discipline is not a corrective for students, but puts them on a pathway toward future punishment and behavior problems (Gerlinger et al., 2021), even with radiating negative effects on classmates who were not excluded (Perry & Morris, 2014).

Although we were unable to empirically assess the potential mediating pathways linking SBLE to increased exclusionary discipline, there are multiple theoretical pathways that merit further examination. First, SBLE may increase the detection of student behaviors without affecting either how students behave or how schools respond to student behaviors (Na & Gottfredson, 2013). Second, SBLE may become involved in the process of administering student disciplinary sanctions, advising school principals and disciplinarians on how they ought to punish students (Kupchik, 2010). Third, SBLE may shift the overall climate of the school to one that is more punitive (Devine, 1996), relying on more formal responses to student behavior (e.g., suspension) rather than informal ones (e.g., corrective conversations with teachers). Fourth, SBLE may cause students to engage in more problem behaviors if students interpret their presence as a sign that behavior problems are normative. Of course, more than one of these explanations may occur simultaneously, and there may be other explanations not listed here.

Additionally, although examining differences in the impacts of SBLE by student race was not a part of this meta‐analysis, an emerging body of evidence continues to point to increases in exclusionary discipline associated with SBLE particularly for Black students (Crosse et al., 2022; Sorensen et al., 2023). Again, given that exclusionary discipline is largely considered a harmful practice for students (Gerlinger et al., 2021), perhaps particularly so for Black students (Fisher et al., 2022), more attention should be given to the racially disproportionate impact of SBLE on the exclusionary discipline of Black students. There is the potential that the increased exclusionary discipline leads to increased likelihood of criminal justice system involvement, which in turn leads to a suite of negative economic, health‐related, and other consequences during adulthood (CITE).

In terms of perception outcomes, overall perceptions of school were not significantly different with or without SBLE. However, among studies that used schools as the unit of analysis, there were greater perceptions of safety among schools with SBLE (although this was based on a model with only seven effect sizes from two studies). None of the models synthesizing learning outcomes were statistically significant, providing no evidence that the presence of SBLE was related to learning outcomes.

### 7.2 Overall completeness and applicability of evidence

The evidence base examining the crime and behavior outcomes associated with SBLE is large and appears to be still growing. This is particularly true of studies that use schools as the unit of analysis. Although there are numerous studies, many of them lack methodological rigor (Petrosino et al., 2012) and there were notably no randomized control trials identified for inclusion in the meta‐analysis. Some of the more recent studies used study designs that permit stronger causal inferences such as interrupted time‐series, instrumental variables, fixed effects, and difference‐in‐differences; this is a notable improvement to the literature that provides more credible estimates than were available even 5 years ago. It is worth noting that several of these studies were based on data from the School Survey on Crime and Safety, and although the effect size estimates derived from these studies were not found to be different from those using other data sources, it would benefit this body of research to have more sources of data that have additional measures of crime and behavior and draw on a different sample of schools. Additionally, there were a limited number of studies that used students as the unit of analysis. Future research may benefit from examining student‐level differences, particularly with an eye toward understanding the experiences of young people who are traditionally marginalized in both education and the criminal legal system, including but not limited to Black, Latino, disabled, and LGBTQ+ students.

Whereas the literature on crime and behavior outcomes was quite robust, the same was not the case for perception and learning outcomes. There were relatively few studies that examined these outcomes, and they were often of poorer methodological quality than those that examined crime and behavior outcomes. This lack of studies makes sense given that perception and learning outcomes are generally considered secondary to the presumed primary outcomes of crime and behavior. Still, if SBLE remain present in schools, it would be useful to better understand their impact on these secondary outcomes. Of particular relevance may be the impact on other aspects of perceived school climate besides feelings of safety. Ethnographic work has linked SBLE to feelings of isolation among students (Nolan, 2011), and also deteriorating student–teacher relationships (Devine, 1996). A few quantitative studies have examined related phenomena (e.g., Fisher et al., 2019; Theriot, 2016), but this evidence base would benefit from more attention to school climate outcomes.

One major limitation of this body of evidence is a general lack of detail about the programmatic elements of SBLE. Very few studies provided specific information about the goals and activities of SBLE programs, making it difficult to assess the extent to which certain elements of SBLE programs may contribute more or less to shaping a variety of outcomes. Although we intended to examine differences across SBLE programs of different types, not enough data about the programming was present in the literature to permit such analyses. Given this limitation in the primary studies, this meta‐analysis can be understood as an analysis of the impacts of the mere presence of SBLE.

Still, it is unclear how and the extent to which different programmatic elements of SBLE programs would shape outcomes differently. Some evidence suggests that as SBLE take on more of a law enforcer role, they respond to student behavior problems with more formal sanctions as opposed to SBLE who identify more as educators and mentors (McKenna & White, 2018). That said, law enforcement activities are often among the most common activities of SBLE (Coon & Travis, 2012). Moreover, research has begun to question the extent to which law enforcement and non‐law enforcement roles can really be separated; one qualitative study found that even as officers describe their non‐law enforcement roles, they articulated them in terms of law enforcement goals and logics (Higgins et al., 2022). A set of studies included in this review examined different arrangements of roles of SBLE, assessing the extent to which officers’ engagement in various activities might differently shape outcomes related to crime, behavior, and responses to student behavior (Devlin & Fisher, 2021; Devlin & Gottfredson, 2018; Fisher & Devlin, 2020). Although these studies found somewhat different patterns of outcomes associated with different SBLE roles, it was not clear that one approach was obviously superior to the others in terms of both reducing problem behaviors and student punishment. So, although it is true that the evidence synthesized in this review lacked important detail about the programmatic elements of SBLE implementation and use, evidence from other research calls into question how important these differences might be.

In terms of generalizability, the vast majority of the studies were set in the United States, so the findings may not generalize beyond that country. Other countries have different cultures around both education and law enforcement, and comparative studies may help elucidate the extent to which these differences shape outcomes associated with SBLE. Additionally, these findings pertain to secondary schools, and the findings may not generalize to preschools, elementary schools, or institutions of higher education. Finally, the measures of the dependent variables included in the analysis (i.e., crime and behavior, perception, and learning outcomes) are based on prior researchers’ measurement and should not be taken to encompass other constructs that may be included within any of those three categories.

### 7.3 Quality of the evidence

One of the major limitations of this review is the quality of the evidence. Although studies in recent years have used increasingly sophisticated study designs and identification strategies that permit stronger causal inferences, many of the studies included here do not have sufficient methodological rigor to permit strong causal inferences. As such, the findings presented here should be interpreted as correlational rather than causal. However, it is noteworthy that in the moderator analyses that examined methodological differences, no significant differences were found, perhaps indicating that the less rigorous studies have come to similar conclusions as the more rigorous ones.

It is particularly noteworthy that there were no randomized control trials included in this review. This is likely because the use of SBLE is highly politicized (Nolan, 2015; Turner & Beneke, 2020) and emotional (Madfis, 2016), and may be difficult and impractical to gain support from practitioners to randomize schools or districts to receive SBLE. Researchers studying SBLE would benefit from continuing to use some of the more rigorous study designs used in the set of studies reviewed here, including time‐series designs (Gottfredson et al., 2020), instrumental variable approaches (Owens, 2017; Weisburst, 2019), and difference‐in‐differences designs that incorporate multiple measures before and after SBLE implementation. Although there were very few studies that met these criteria in the current review, evidence about the impacts of SBLE based on rigorous study designs continues to accrue, with at least two studies becoming available since the we conducted final search for this review (Crosse et al., 2022; Sorensen et al., 2023). Although this meta‐analysis provides evidence from studies with a range of methodological rigor—and finds general consistency when examining differences based on methodological characteristics—these more rigorous studies permit stronger causal inferences than those with weaker designs. Moreover, in a political climate that has recently seen more widespread critiques of SBLE, even calling for their removal, it will be important to use rigorous methods to investigate what happens to school and student outcomes when SBLE is removed.

Additionally, the finding about SBLE being associated with increased perceptions of safety should be interpreted with caution. Although the finding was statistically significant, it was based on a meta‐analysis of only two studies that provided a total of seven effect sizes. Moreover, these two studies (McKay et al., 2006; Stokes et al., 1996) used fairly weak study designs that do not permit strong causal inferences. Future studies would benefit from applying more rigorous methods to examining the impact of SBLE on students’ feelings of safety at school.

### 7.4 Potential biases in the review process

As with most empirical research, this review included many researcher choices that may have introduced bias into the review process. For example, we chose to aggregate all effect sizes that fell under each of the three broad topics (i.e., crime and behavior, perception, and learning outcomes) and identify common groupings within each category. Other researchers may have made different decisions about what effect sizes should and should not be grouped together, and this may shape the findings and conclusions of the study.

We also opted to include a wide range of studies rather than imposing strict methodological criteria that would have led to only those studies with the strongest internal validity to be included. As such, some effect sizes are likely more trustworthy than others not in terms of the precision of the estimate, but because of the design of the study from which they were derived. Although we tested for moderating effects of multiple study design features, other researchers may have made different choices in this vein.

Additionally, as noted, because of personnel limitations associated with the multiple literature search and screening processes, not all screening and data extraction was completed by two coders. This could have introduced bias through human error that might have been detected if another coder was available.

Finally, we as a research team acknowledge our own cultural embeddedness that shaped a variety of choices we made during the process of conducting this review, ranging from the conception of the study to particular analytic choices to the perspectives we chose to highlight in this narrative. Other research teams with different enculturation and perspectives may have conducted this review differently, and we encourage replication by future researchers.

### 7.5 Agreements and disagreements with other studies or reviews

To our knowledge, there have been two prior meta‐analyses related to SBLE. The findings from this study have some similarities and differences with these two meta‐analyses. For instance, one prior meta‐analysis of longitudinal research found that high schools with school resource officers—one type of SBLE—experienced increases in exclusionary discipline associated with the implementation of the officers (although, notably, the model using cross‐sectional studies showed null results; Fisher & Hennessy, 2016). This aligns closely with the current study's finding that associates SBLE with increased exclusionary discipline.

Additionally, this study's findings align with another meta‐analysis that found no significant relationship between the presence of a law enforcement officer or security guard at school and school violence and victimization (Turanovic et al., 2019). This is similar to the current study's findings that do not find a significant link between SBLE and measures of violence, or weapon‐related outcomes. It is notable that the current study's finding linking SBLE to greater crime and behavior problems was driven largely by the increase in school discipline, a measure that was not included in the Turanovic et al. (2019) study.

## 8 AUTHORS’ CONCLUSIONS

### 8.1 Implications for practice

Most schools in the United States—and many schools in other countries where data are not systematically collected—use some form of SBLE, at least implicitly to make the school safer. However, this study's findings indicate that SBLE presence is linked with an increase in school crime and behavior problems. Moreover, when investigating more specific types of crime and behavior problems, this study found no impact on outcomes related to criminal justice system contact, violence, to substance use or possession, or weapon use or possession. If the intent of SBLE is to prevent crime and delinquency in schools, this study found no evidence that this intent is being accomplished. Instead, consistent with a more critical literature that focuses on the harms of SBLE, this study found that SBLE presence was associated with increases in school discipline. This is particularly noteworthy given the robust literature that has linked school discipline to a variety of adverse consequences, including increased delinquency and contact with the criminal justice system (Gerlinger et al., 2021).

Given these findings, practitioners are likely to benefit from reconsidering their use of SBLE. Momentum for removing SBLE has been growing in the United States, and this study's findings imply that removing SBLE is likely to reduce schools’ use of exclusionary discipline, and perhaps even decrease the amount of crime that happens in school, although it is difficult to disentangle the amount of crime that *happens* from the amount of crime that is *detected*. The benefits associated with this are unclear—although it may be valuable to detect more crimes, if those are highly subjective offenses like disorderly conduct that are recategorized and responded to as crimes (rather than school disciplinary events), it may have harmful consequences for students (Theriot, 2009). Still, given the financial costs of maintaining SBLE presence, the lack of clear benefits identified in this meta‐analysis suggests that money is likely better spent elsewhere. Research has not examined in any depth the extent to which other school personnel may reduce school crime, but this may be a fruitful line of inquiry.

For schools that do choose to retain SBLE, it is unclear whether there are best practices that will enhance their effectiveness while limiting their negative consequences. Certainly, there are recommended strategies based on experience (e.g., see Petrosino et al., 2020). A few studies have investigated how implementing SBLE with different arrangements of roles is related to crime and behavior outcomes at school, but these studies’ findings are inconsistent and point to no real definitive conclusion about whether and how the roles of SBLE might make a difference (Devlin & Fisher, 2021; Devlin & Gottfredson, 2018; Fisher & Devlin, 2020). Although some scholars and practitioners have pointed to the importance of choosing the “right” officer, having memoranda of understanding in place, giving formal training, and not having officers be part of school discipline (Finn et al., 2005; Rosiak, 2016) these strategies have not been thoroughly evaluated, and studies show these efforts may be difficult to achieve and even counterproductive (Bolger et al., 2019; Curran et al., 2021). We advise those jurisdictions using SBLE to study their programs to ensure they are not harming the students and staff and are having the appropriate impact on the school community. Schools and communities that want to employ SBLE should look to innovate with current SBLE models and then test to see if the new type of strategy had desired impacts.

If schools choose to divest from SBLE they are likely to benefit from using those funds on promising evidence‐based practices for reducing crime and violence in schools. One promising approach is to invest in school climate improvement. For example, increasing students’ sense of belonging at school and feelings of connection to peers and adults in the school is an effective strategy for preventing problematic behaviors (Gregory et al., 2012; Hirschi, 1969; Payne et al., 2003). Research in the last decade has increasingly focused on an authoritative school climate, which prioritizes both support and structure in the form of (a) strong interpersonal relationships between students and school personnel, and (b) clear, fair, and consistently enforced rules. This approach has shown benefits for a variety of outcomes related to crime and behavior (Berg & Cornell, 2016; Cornell et al., 2015; Crowley et al., 2019; Fisher et al., 2017; Gerlinger & Wo, 2016; Huang et al., 2021).

### 8.2 Implications for research

The findings from this meta‐analysis have implications for research on SBLE. First, as noted, methodologically rigorous studies are needed to address the issue of selection bias that is common in this literature. Using the receipt of grant funding for SBLE as an instrumental variable has been a promising approach, as has estimating pre‐trends using many time points. Further research in this vein will be a useful contribution to this literature.

Second, given the pessimistic findings from this study and the growing movement toward removing SBLE, research is needed on what happens when SBLE is removed. Studies of SBLE removal should focus on measurable outcomes such as school crime, discipline, and perceptions of safety, but also procedural and cultural outcomes that may be better addressed through qualitative research. How do school administrators compensate for their loss of a resource in the school? What new strategies or processes emerge for handling crime or misbehavior? How does the relationship between schools and police change as a result? Although this study's findings are suggestive that removing SBLE may have benefits for students and schools, this is only one step among many that schools might take, and it is critical to understand both the process of and outcomes associated with removing SBLE.

Third, research should focus more on issues of equity associated with SBLE implementation, use, and removal. There is both theoretical reasoning (Hirschfield, 2008; Kupchik & Monahan, 2006) and empirical evidence in the US setting (Crosse et al., 2022; Fisher et al., 2020; Homer & Fisher, 2020; Weisburst, 2019) to suggest that SBLE have disproportionate impacts on traditionally marginalized students—especially Black students. More evidence is needed to better understand the nature of this problem, and to expand the analysis to other traditionally marginalized groups of students including students with disabilities, LGBTQ+ students, undocumented students, and others.

Fourth, a greater focus on the role of context is likely to be important. There is evidence from the United States that the roles and perspectives of SBLE are contingent on their school's context (Fisher et al., 2020; Lynch et al., 2016). However, relatively little research in this body of literature has focused explicitly on the role that characteristics of school and community contexts might play in the impacts of SBLE. Further attention to this would likely benefit research, policy, and practice alike.

## CONTRIBUTIONS OF AUTHORS

Benjamin W. Fisher, Anthony Petrosino, Hannah Persson, Sarah Guckenburg, Trevor Fronius, Ivan Benitez, and Kevin Earl contributed to the writing and revising of this review. Ivan Benitez, Hannah Persson, Kevin Earl, and Anthony Petrosino conducted the search strategy. Benjamin W. Fisher, Hannah Persson, Kevin Earl, Sarah Guckenburg and Trevor Fronius coded the relevant reports and performed the reliability check. Benjamin W. Fisher conducted the quantitative meta‐analyses. Anthony Petrosino will be responsible for updating this review as additional evidence accumulates and as funding becomes available.

## DECLARATIONS OF INTEREST

The authors declare no conflicts of interest.

## PLANS FOR UPDATING THE REVIEW

We plan to update this review every 3 years in accordance with Campbell policy, and contingent on resources.

## DIFFERENCES BETWEEN PROTOCOL AND REVIEW

The eligibility criteria differed slightly between the protocol and the review. Specifically, the protocol specified that pre–post study designs without a comparison group would only be included if there were many data points both before and after the implementation of SBLE (i.e., an interrupted time series design). However, because interrupted time series designs are so rare in this literature, and to cast a wider net that permitted more studies to be included, we allowed for pre‐post designs without a comparison group that had as few as one observation before and after the implementation of SBLE.

A second change to the eligibility criteria was in regard to school administrator perceptions of the outcomes. The protocol called for excluding these studies, but the review included them. Many of the most rigorous studies in this literature rely on survey data from school administrators (especially from the SSCOS data), and we wanted to ensure that these studies were included in the review because of their salience in the field and relative strong study designs relative to other included studies. Nevertheless, we did not find that the weighted mean effect sizes of studies using the SSOCS data were significantly different from those using other data.

We also revised the analytic strategy. The protocol called for using a single “best” effect size from each study so that each study would provide only one effect size. The review, however, used multiple effect sizes from each study (as they were available). This change was made to avoid losing data or making arbitrary choices about which effect sizes to include and exclude. Additionally, the availability of estimation methods that accommodate dependent effect sizes has expanded greatly since the protocol was written, rendering the strategy written there somewhat outdated.

The protocol proposed using four moderators to examine heterogeneity, including intervention type, school level, research design (experimental vs. quasi‐experimental), and whether the report was published. The first three moderators listed here were either unavailable in the vast majority of reports, or in the case of school level showed no variability. As such, we added additional moderators (as noted above in the Method section) in an effort to explain the heterogeneity.

## SOURCES OF SUPPORT

### 1 Internal support

We appreciate in‐kind support from WestEd.

### 2 External support

This study was funded in part by the Campbell Collaboration Education Group (University of Arkansas).

## Supporting information

Supporting information.

## Footnotes

1 Note that there was not enough information in the retrieved studies to respond adequately to this question.

2 Although, please note that the reports retrieved in the 2020 update were only reviewed by one coder.
