# Supplementary material for: School‐based law enforcement strategies to reduce crime, increase perceptions of safety, and improve learning outcomes in primary and secondary schools: A systematic review
Source: Campbell Syst Rev. 2023 Nov 8;19(4):e1360. doi: 10.1002/cl2.1360 (PMC10630714; doi:10.1002/cl2.1360)
Supplement: Supplementary file 1 — Supporting information. [file CL2-19-e1360-s001.docx]

**Appendix A – Electronic Databases Searched**

**Databases Included in Original Search in 2009**

| Database | Platform |
| --- | --- |
| Academic Search Premiere | EBSCO |
| The Alcohol and Alcohol Problems Science Database (ETOH) |  |
| American Periodical Series Online | PROQUEST |
| Bibliography of Nordic Criminology/Criminal Justice in Denmark |  |
| British Public Library Integrated Catalog |  |
| Canadian Evaluation Society Grey Literature | https://evaluationcanada.ca/search |
| California Peace Officers Standards and Training Law Enforcement Archives | https://post.ca.gov/ |
| Chalk’s E-Library |  |
| Chicano Database |  |
| Claseperiodica Abstracts (Caribbean and Latin America) |  |
| Cochrane Library: Cochrane Central Controlled Trial Register | EBSCO |
| Cochrane Library: Cochrane Health Technology Assessment Database |  |
| Cochrane Library: National Health Service Economic Evaluations |  |
| Conference Papers Index |  |
| Criminal Justice Abstracts | EBSCO |
| Database of African theses and Dissertations |  |
| Database of Research in International Education |  |
| EBSCO Master File | EBSCO |
| EBSCO Mega-file | EBSCO |
| EBSCO Military and Government Collection | EBSCO |
| EBSCO SOCINDEX | EBSCO |
| Econlit | EBSCO |
| ECONPAPERS |  |
| Education Administration Abstracts |  |
| Education Resources Information Center (ERIC) | EBSCO |
| Education Full-Text | EBSCO |
| Education Retro Index |  |
| Educators Reference Complete InfoTrac |  |
| Expanded Academic ASAP Plus |  |
| Family and Society Studies Abstracts | EBSCO |
| First Search OCLC |  |
| General OneFile Infotrac |  |
| Google | https://www.google.com |
| Google Scholar | https://www.scholar.google.com |
| Homeland Security Digital Library | EBSCO |
| Index to Current Urban Documents |  |
| Index to Foreign Legal Periodicals |  |
| International Bibliography of the Social Sciences | PROQUEST |
| ISI Web of Knowledge/Social Science Citation Index |  |
| JRSA ISAR |  |
| JSTOR | EBSCO |
| Medline | EBSCO |
| National Bureau of Economic Research Working Papers | <https://www.nber.org/papers?page=1&perPage=50&sortBy=public_date> |
| National Criminal Justice Reference Service | <https://www.ncjrs.gov/whatsncjrs.html%5C> |
| Ovid Full-Text Journals and Ovid Books |  |
| Policy Archive |  |
| Policy File |  |
| ProQuest Dissertations | EBSCO |
| Psychology and Behavioral Sciences Collection | EBSCO |
| Psychological Abstracts (PsycInfo) | EBSCO |
| Public Affairs Information Service (PAIS) | EBSCO |
| Public Affairs Information Service (PAIS) International |  |
| Race Relations Abstracts | EBSCO |
| Sage Criminology Full-Text | EBSCO |
| Selected Periodicals Index Online |  |
| Social Service Abstracts | EBSCO |
| Social Work Abstracts | EBSCO |
| Sociological Abstracts (Sociofile) | PROQUEST |
| SSRN Electronic Library | https://www.ssrn.com/index.cfm/en/ |
| Theses Canada |  |
| UK and Ireland Dissertations and Theses |  |
| Urban Studies Abstracts | EBSCO |
| World Bank Documents |  |
| Worldwide Political Abstracts | PROQUEST |

**Databases Included in Most Recent Search in 2020**

| Database | Platform |
| --- | --- |
| Academic Search Premiere | EBSCO |
| American Periodical Series | PROQUEST |
| Canadian Evaluation Society Grey Literature | https://evaluationcanada.ca/search |
| California Peace Officers Standards and Training Law Enforcement Archives | https://post.ca.gov/ |
| Cochrane Library: Cochrane Central controlled Trial Register | EBSCO |
| Criminal justice abstracts | EBSCO |
| EBSCO Educational administration abstracts | EBSCO |
| EBSCO Masterfile | EBSCO |
| EBSCO social index | EBSCO |
| Econlit abstracts | EBSCO |
| Education Resources Information Center (ERIC) | EBSCO |
| Education full text | EBSCO |
| Family and society studies | EBSCO |
| Google | https://www.google.com |
| Google Scholar | https://www.scholar.google.com |
| Homeland Security Digital Library | EBSCO |
| International Bibliography of the Social Sciences | PROQUEST |
| JSTOR | EBSCO |
| Medline abstracts | EBSCO |
| National Bureau of Economic Research Working | <https://www.nber.org/papers?page=1&perPage=50&sortBy=public_date> |
| National Criminal Justice Reference Service | <https://www.ncjrs.gov/whatsncjrs.html%5C> |
| ProQuest Dissertations | EBSCO |
| Psychology and behavioral sciences collection | EBSCO |
| Psychological abstracts (PsycInfo) | EBSCO |
| Public Affairs Information Services (PAIS) | EBSCO |
| Race relations | EBSCO |
| Sage Criminology Full text | EBSCO |
| Selected Periodicals Index Online |  |
| Social services abstracts | EBSCO |
| Social Work Abstracts | EBSCO |
| Sociological abstracts | PROQUEST |
| SSRN Electronic Library | https://www.ssrn.com/index.cfm/en/ |
| Urban studies abstracts | EBSCO |
| Worldwide political abstracts | PROQUEST |

*Note*. Where possible, all searches were restricted to the date range of January 1, 2014 through June 1, 2020.

**Appendix B – Data Extraction Instrument**

C2 Education Review: Policing Schools

CODING INSTRUMENT

**Coder:**

- Sarah Guckenburg
- Trevor Fronius
- Anthony Petrosino
- Alexis Stern
- Other ____________________________________________

**Citation for Primary Document**: ________________________________________________________________________________________________________________________________________________________________________________________________________________________________________________________________________________________________

# I. RESEARCHER AND STUDY CHARACTERISTICS

# What year was the document was published? _________________________________

# What was the type of primary document?

- - - - - Book
        - Book Chapter
        - Government Report
        - Journal (peer reviewed)
        - Open-access electronic journal
        - Dissertation
        - Unpublished (tech report, conference paper)

**How many documents were considered in coding this study?** ____________________

**What state or country did the study take place?**

____________________

**What was the setting for the study?**

____________________

**What other information was provided on the context for the evaluation? (This can include the rationale for the study; more about the setting; anything to help us learn more about the context)**

**____________________________________________________________________________________________________________________________________________________________________________________________________________________________**

**Indicate any inclusion/exclusion criteria for the units of analysis in the study**

_______________________________________________________________________________________________________________________________________________________________________________________

**Who funded this study?** (Should be in the acknowledgment section) ________________________________________________________________________________________________________________________________________________

**What were the relationships of the authors to the development and/or implementation of the treatment?**

________________________________________________________________________

**II. STUDY METHODS AND METHODOLOGICAL QUALITY**

**Was random assignment used to assign groups?** **(Yes/No)**

**At what level was randomization conducted? _________________________________**

**How was the randomization specifically done? _______________________________________________________**

**______________________________________________________________________________________________________________**

**Were any randomization problems (e.g., contamination, crossovers) noted?** (Yes/No)

*If yes, please detail those problems below:*

_______________________________________________________________________________________________________________________________________________________________________________________

How did investigators deal with randomization problems?

_______________________________________________________________________________________________________________________________________________________________________________________

**If random assignment was not used, what quasi-experimental method was used to equate groups? (e.g., matched comparison schools; post-hoc statistical matching of individuals; regression discontinuity; propensity scores; etc.)**

________________________________________________________________________________________________________________________________________________________________________________________________________________________________________________________________________________________________

**Were any problems with non random assignment noted? (Yes/No)

If so, what were they?**

**________________________________________________________________________________________________________________________________________________________________________________________________________________________**

**How did investigators deal with non-random assignment problems?**

**_____________________________________________________________________________________________________________________________________________________________________**

**Where did comparison group come from?**

__________________________________________________________________________________________________________________________

**At what level was non-random assignment made?** _____________________________

**Were any substantive differences in pretests of group equivalence noted?** **(Yes/No)**

*If yes, please detail those differences below:*

_______________________________________________________________________________________________________________________________________________________________________________________

**Was overall attrition problem from originally assigned sample noted?** (Yes/No)

**Was differential attrition noted?** (Yes/No)

*If yes, please detail those problems below (especially the magnitude of attrition, both from original sample and differentially between treatment and control groups):*

____________________________________________________________________________________________________________________________________________________________________________________________________________________________________________________

**How was attrition dealt with by investigators?** _______________________________________________________________________________________________________________________________________________________________________________________

**III. INTERVENTION AND CONTROL CONDITIONS**

**Number of groups in the study:** ________________

**Rationale for selecting intervention and control contrast if multiple groups:**

________________________________________________________________________________________________________________________________________________

**List excluded study groups with brief description:** _______________________________________________________________________________________________________________________________________________________________________________________

**Describe the intervention below, with particular attention to the “dosage” of the treatment:**

_______________________________________________________________________________________________________________________________________________________________________________________

***How many cases were randomized or assigned to this group?*** __________________

**Were there any program implementation problems described by investigators?** (Yes/No)

***Detail fidelity problems below:***

_______________________________________________________________________________________________________________________________________________________________________________________

**Please detail program theory (**or mechanisms for why it should work. Also include any post-hoc information from study on why the program worked or did not work**):**

_______________________________________________________________________________________________________________________________________________________________________________

**What is the control or comparison condition?**

- No Treatment Group
- Wait-List Control
- Treatment as Usual Group
- Placebo
- Lesser but Innovative Treatment

**Describe the control or comparison condition (including “dosage” if applicable):**

_______________________________________________________________________________________________________________________________________________________________________________________

*How many cases were randomized or assigned to this group?* ___________________

**IV. PARTICIPANTS IN THE STUDY**

**Type of school**

_______________________________________________

**Age/school level/grade** _______________________________________________

**Percentage of participants that were female** ________________________________

**Percentage of participants that were white** ___________________________________

**Poverty/SES** ___________________________________________________________

**Other data on participants:**

__________________________________________________________________________________________________________________________

**V. OUTCOMES**

**(see next page)**

Include all data on treatment and control, including results, sample sizes used in analysis, the statistical technique, whether regression-adjusted or not, (and if so, what controls were used), statistical significance and probability level.

| **Outcome** | **First Effect (Months:________)** | **Last Effect (Months:________)** |
| --- | --- | --- |
| Crime/Behavior |  |  |
| Perceptions |  |  |
| Learning Outcomes |  |  |
| Other |  |  |

**Please detail all subgroup effects below:**

________________________________________________________________________________________________________________________________________________________________________________________________________________________________________________________________________________________________________________________________________________________________________________________________________________________________________________

**Provide any information on qualitative data in the study:**

________________________________________________________________________________________________________________________________________________________________________________________________________________________________________________________________________________________________________________________________________________________________________________________________________________________________________________________________________________________________________________________________________________________________________________________________

**Please detail all cost/economic information below:**

________________________________________________________________________________________________________________________________________________________________________________________________________________________________________________________________________________________________________________________________________________________________________________________________________________________________________________________________________________________________________________________________________________________________________________________________

**ANY OTHER COMMENTS ON THE PROGRAM OR EVALUATION (use bullets)**
